# The global and regional burden and trends of migraine from 1990 to 2021: Global Burden of Disease Study 2021

**DOI:** 10.3389/fneur.2025.1686288

**Published:** 2025-11-03

**Authors:** Ying Lu, Quan-yao Li, Lu Gan, Yi You, Chang-de Wang, Zhao-wei Guo, Jun Shi, Xiao-ying Liu

**Affiliations:** ^1^Shanghai University of TCM Shanghai TCM-Integrated Hospital, Shanghai, China; ^2^Jing’an District, Shanghai (Medical School of Tongji University) Pengpu Xincun Community Health Care Center Internet Hospital, Shanghai, China; ^3^Shanghai Fourth People’s Hospital Affiliated to Tongji University, Shanghai, China

**Keywords:** migraine, disease burden, frontier analysis, trends, GBD 2021

## Abstract

**Background:**

Migraine is a common disabling neurological disorder that affects the quality of human daily life and mental health, potentially leading to disability and imposing a substantial disease burden. There is currently a lack of literature analysis on the global burden and changing trends of migraines. Therefore, this study is of great significance for the development of effective management and prevention strategies for migraine by analyzing the global burden of migraine and its changing trends.

**Methods:**

We obtained data on the incidence, prevalence, and disability-adjusted life years (DALYs) of migraine by region and year from the Global Burden of Disease Study 2021 (GBD 2021). The estimated annual percentage change (EAPC) and 95% confidence interval (CI) were used to evaluate the time trend of disease burden, and factors that may affect the EAPC were analyzed. The burden of migraine was analyzed based on the Socio-demographic Index (SDI). Frontier analysis was used to visually demonstrate the burden of development levels in various regions, and the trends of migraine incidence, prevalence, and DALYs over the next decade were predicted.

**Results:**

In 2021, the global number of prevalent cases, incident cases, and DALYs for migraine were 1.158 billion, 90.18 million, and 43.38 million, compared with 1990, the percentage changes were 58, 42, and 58%, respectively. In 2021, the age-standardized incidence rate (ASIR), age-standardized prevalence rate (ASPR), and age-standardized DALY rate (ASDR) of migraine were the highest in areas with high SDI. Its prevalence, incidence, and DALYs were 15365.1, 1222.5, and 573.6 per 100,000 people. From 1990 to 2021, the ASDR showed an upward trend globally and in all regions, among which ASIR and ASPR in global, China, East Asia, high, high-middle SDI regions were on the rise, while they showed a downward trend in low, low-middle SDI regions. Frontier analysis showed that areas with high SDI generally had greater potential for improvement. We used a predictive model to estimate that ASIR, ASPR, and ASDR of migraine were all on the rise.

**Conclusion:**

From 1990 to 2021, the global burden of migraine has increased significantly, especially in middle SDI region. This study highlights the importance of tailored interventions aimed at addressing migraine and thus contributing to the achievement of the Sustainable Development Goals set by the World Health Organization.

## 1 Introduction

Migraine is a common chronic neurovascular dysfunction disease, ranking as the third most disabling condition in the nervous system (1). It is characterized by recurrent unilateral or bilateral pulsatile pain, sensitivity to sound and light stimuli, accompanied by nausea, vomiting, and other symptoms. It has a high recurrence rate, affecting the quality of human daily life and mental health, and can even lead to disability in severe cases. Migraine demonstrates established comorbidities with anxiety, depression, and sleep disorders, while significantly elevating the risk of cognitive dysfunction and cardiovascular/cerebrovascular diseases (2). Furthermore, evidence confirms associations with increased susceptibility to asthma, stroke, and other pain-related conditions (3, 4). These factors collectively establish migraine as a substantial public health challenge that inflicts considerable patient suffering and imposes major socioeconomic burdens (3).

The Global Burden of Disease Study 2021 (GBD 2021) provides the latest data on migraine diseases covering 204 countries and regions worldwide from 1990 to 2021 (5). This latest iteration incorporates expanded data sources and methodological refinements that enhance estimation accuracy. While the GBD 2019 report highlighted migraine’s significant contribution to disability-adjusted life years (DALYs), particularly among the 10–49 age group where it represents a leading cause of disability (6). For the diagnosis and treatment of migraine, the ideal situation is to follow internationally recognized recommendations and establish a layered medical service provision structure. However, in reality, significant obstacles are encountered. In most countries around the world, headache patients fail to receive standardized treatment due to insufficient medical system resources. Furthermore, the COVID-19 pandemic has further exacerbated these inequalities (7). Therefore, this study analyzes the incidence, prevalence, and DALYs of migraine through GBD 2021, predicts its trend, and aims to comprehensively evaluate the global disease burden of migraine from 1990 to 2021. Compared with previous GBD studies, this study integrates more available data sources and provides more accurate estimation results through improved methodology. It is expected to serve as an important extension and supplement to the aforementioned research, helping healthcare workers to have a deeper understanding of migraine and providing a scientific basis for policymakers to improve resource allocation and develop effective health strategies.

Building upon previous important work documenting migraine burden, the evolving data landscape and global context necessitate continued assessment. The Global Burden of Disease Study 2021 provides updated estimates through 2021, capturing the initial phase of the COVID-19 pandemic—a crucial period for understanding this global health crisis’s impact on chronic disease management, including migraine (8). While confirming established patterns of migraine burden from earlier reports, our study extends this work by systematically examining heterogeneity in disease burden across Socio-demographic Index (SDI) regions and projecting future trends using frontier analysis. Through extended temporal analysis (1990–2021) and SDI-stratified interpretation, our findings provide updated epidemiological evidence with enhanced granularity to inform targeted migraine prevention and control strategies globally.

## 2 Methods

### 2.1 Disease definition and data sources

According to the GBD 2021, migraine is defined as a disabling primary headache disorder characterized by recurrent moderate to severe pulsatile unilateral headache. In GBD 2021, we do not distinguish between migraine with aura and migraine without aura because most epidemiological studies only report overall migraine data. If a patient’s symptoms meet the five diagnostic criteria proposed by the International Classification of Headache Disorders, Third Edition (ICHD-3), they can be diagnosed with migraine. According to the 9th and 10th editions of the International Classification of Diseases (ICD), migraine is represented by codes 346–346.93 and G43-G43.919, respectively (5).

This study used anonymized data from the GBD 2021, a comprehensive database covering the impact of 371 diseases, 88 risk factors, and injuries in 5 Socio-demographic Index (SDI) groups and 204 countries and regions. The information is available at the following URL: http://ghdx.healthdata.org/gbd-results-tool. Detailed information on relevant data, methods, and statistical modeling can be found in previous reports (8). We obtained migraine incidence, prevalence, and DALYs data by region and year from the GBD 2021. The estimated annual percentage change (EAPC) and 95% confidence interval (CI) were used to evaluate the time trend of disease burden. The migraine related burden was analyzed based on the Socio-demographic Index (SDI), a comprehensive indicator that measures education, economy, and fertility levels, emphasizing the interconnection between social development and population health outcomes. Frontier analysis was further applied to evaluate the relationship between migraine burden and socio-demographic development. Subsequently, we extracted the corresponding predicted population to forecast the global migraine age-standardized incidence rate (ASIR), age-standardized prevalence rate (ASPR), and age-standardized DALY rate (ASDR) for the next 10 years.

### 2.2 Statistical analysis

This study calculated the EAPC to describe the ASR trend of migraine burden. If both the EAPC value and the lower limit of its 95% confidence interval are greater than 0, it indicates an upward trend in ASR. If these values are less than 0, it indicates a downward trend. If the EAPC is equal to 0, it indicates a constant trend. All data analyses in this study were performed in R software (version 4.2.2) and RStudio, and a *p*-value <0.05 was considered statistically significant.

## 3 Results

### 3.1 Global level

**Figure 1:**
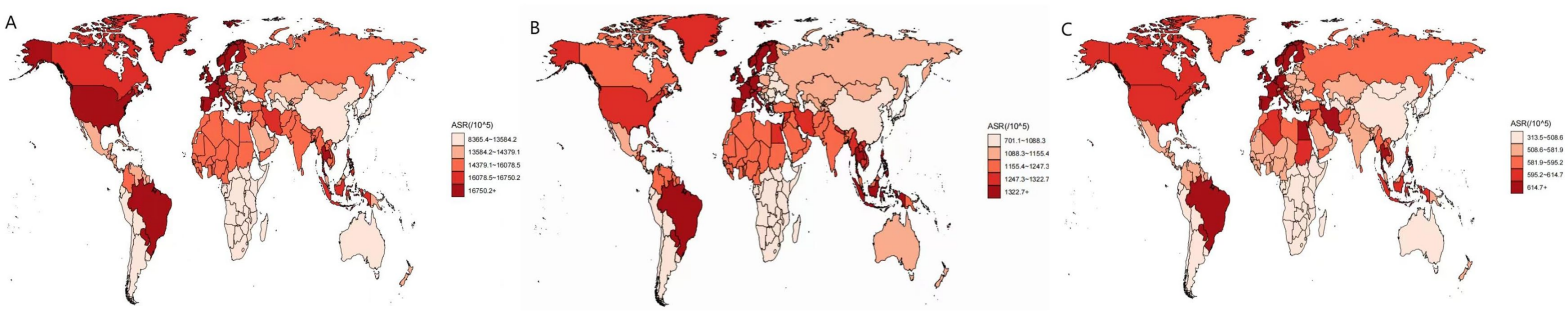
Global map of ASIR, ASPR, and ASDR of migraine for 204 countries and regions in 2021 (**A**: ASIR, **B**: ASPR, **C**: ASDR).

Globally, there has been a significant increase in the number of prevalent cases, incident cases, and DALYs for migraine. For instance, migraine prevalent cases rose from 732.56 million in 1990 to 1.15843 billion in 2021, representing a 58% increase. The number of incident cases rose from 63.5 million in 1990 to 90.18 million in 2021, representing a 42% increase. Similarly, DALYs cases rose from 27.41 million in 1990 to 43.38 million in 2021, also a 58% increase (Tables 1–3 and Figure 1). The global prevalence rate, incidence rate, and DALYs rate of migraine have all climbed from 1990 to 2021, with EAPC of 0.05 (95% CI: 0.04–0.06), 0.05 (95% CI: 0.03–0.07), and 0.04 (95% CI: 0.04–0.04) respectively. This indicates a persistent and profound increase in the global burden of migraine.

**Table 1:** Global and regions prevalence of migraine from 1990 to 2021.

Number	Location	Number (1990)	ASR per 100,000 (1990)	Number (2021)	ASR per 100,000 (2021)	EAPC (CI)
1	China	133474536.5 (114199443.7–153482597.7)	10948.5 (9428.8–12586.1)	184752280.1 (160836524.7–213633958.3)	11777.5 (10137.6–13538.6)	0.24 (0.23–0.24)
2	East Asia	138666855.2 (118605236.2–159459078.7)	10993.2 (9469–12636.8)	191704023.9 (166846937.8–221713278.7)	11798.4 (10162.5–13,567)	0.23 (0.23–0.23)
3	Global	732564462.7 (624559243.9–847058436.3)	14027.6 (12063.4–16078.1)	1158432823.8 (995861966.4–1331312506.1)	14246.5 (12194.1–16378.7)	0.05 (0.03–0.07)
4	High SDI	142911565.2 (124064905–163809089.4)	15286.9 (13196.4–17504.2)	175424403.7 (153279198.8–201,895,737)	15365.1 (13250.3–17765.4)	0.01 (−0.13–0.14)
5	High-middle SDI	146640057.8 (125805580.8–168593873.1)	13250.6 (11372.4–15230.6)	191802656.3 (167163077.2–220,197,711)	13502.7 (11610.1–15484.7)	0.06 (0.06–0.07)
6	Low SDI	54342303.5 (45978743.5–63691837.1)	12809.1 (10955.4–14788.5)	129654870.3 (109563874.3–152432455.6)	12,809 (10909.3–14754.4)	0 (−0.03–0.02)
7	Low-middle SDI	157234783.9 (133322760.3–182843387.7)	14823.8 (12712.9–17056.2)	288676927.2 (246439632.7–332108194.8)	14,787 (12677.1–16,979)	−0.01 (−0.05–0.03)
8	Middle SDI	230715534.9 (195772574.5–267365243.4)	13590.8 (11682.7–15580.9)	371941648.1 (319831620.3–426895953.9)	14344.2 (12233.4–16477.3)	0.18 (0.16–0.19)

**Table 2:** Global and regions incidence of migraine from 1990 to 2021.

Number	Location	Number (1990)	ASR per 100,000 (1990)	Number (2021)	ASR per 100,000 (2021)	EAPC (CI)
1	China	11518097.6 (10091942.1–13156841.9)	917.3 (808.4–1,037)	13047220.7 (11597731.5–14698852.1)	975.6 (862.3–1102.1)	0.2 (0.19–0.2)
2	East Asia	11956378.8 (10465336.6–13649232.3)	921 (810.9–1041.6)	13524742.6 (12000699.7–15250401.1)	976.9 (863.3–1,104)	0.19 (0.19–0.19)
3	Global	63496590.8 (55194751.5–72208003.4)	1136.9 (995.1–1287.8)	90183386.9 (78857600.5–101838162.5)	1153.2 (1006.1–1304.5)	0.05 (0.04–0.06)
4	High SDI	10193796.2 (8908219.3–11542805.2)	1200.5 (1044.7–1359.7)	11294915.5 (9902278.8–12786995.5)	1222.5 (1070.6–1388.6)	0.05 (−0.08–0.17)
5	High-middle SDI	11546733.7 (10130462.1–13148277.8)	1056.8 (920.6–1201.7)	13060790.8 (11423422.8–14774640.4)	1087.1 (951.7–1233.3)	0.09 (0.06–0.13)
6	Low SDI	5490327.4 (4696368.4–6408793.2)	1047.9 (909–1192.8)	12662944.1 (10811145–14650313.1)	1,046 (905.2–1193.5)	−0.01 (−0.03–0.02)
7	Low-middle SDI	15222630.5 (13175203.1–17497048.4)	1220.6 (1066.8–1377.8)	24656879.9 (21420024.1–27993901.8)	1213.3 (1057.8–1372.5)	−0.02 (−0.06–0.01)
8	Middle SDI	20983717.3 (18289662.7–23872475.4)	1129.4 (987.4–1,276)	28437798.3 (25055734.6–32051775.7)	1173.2 (1027.7–1322.8)	0.13 (0.06–0.2)

**Table 3:** Global and regions DALYs of migraine from 1990 to 2021.

Number	Location	Number (1990)	ASR per 100,000 (1990)	Number (2021)	ASR per 100,000 (2021)	EAPC (CI)
1	China	5028787.5 (767668.5–11262271.4)	413 (66.2–911)	6988198.6 (1133318.7–15186289.3)	443.7 (66.9–971.7)	0.23 (0.23–0.23)
2	East Asia	5223002.7 (792490.7–11671556.1)	414.5 (65.9–912.6)	7248392.4 (1171602.6–15744802.5)	444.3 (66.8–971.8)	0.22 (0.22–0.23)
3	Global	27412196.3 (4076605–60325805.8)	526.8 (83.4–1145.9)	43378889.8 (6732642.2–95079454.1)	532.7 (80.6–1167.7)	0.04 (0.04–0.04)
4	High SDI	5,376,701 (896805.6–11550290.6)	573.4 (91.4–1243.2)	6613112.3 (1212857.1–14047722.3)	573.6 (89.8–1,236)	−0.01 (−0.1–0.08)
5	High-middle SDI	5657198.1 (1090025.3–12056696.2)	511.7 (99.7–1085.8)	7409676.3 (1474226.7–15665836.9)	517.6 (92.9–1103.3)	0.04 (0.01–0.07)
6	Low SDI	1989610.8 (284626.4–4460947.3)	472.4 (76.2–1018.8)	4782245.5 (672592.6–10758550.4)	475.2 (76.4–1031.5)	0.02 (0.01–0.03)
7	Low-middle SDI	5750275.1 (691692.1–12804149.2)	543.9 (73.7–1198.3)	10,617,257 (1368932.8–23440099.6)	544.2 (73.9–1201.9)	0 (−0.03–0.03)
8	Middle SDI	8611314.2 (1117747.6–19168775.6)	508.5 (72.1–1119.5)	13921317.3 (2023382.9–30,544,094)	535.9 (74.8–1178.3)	0.17 (0.15–0.19)

In East Asia, prevalent cases rose from 138.67 million in 1990 to 191.7 million in 2021, representing a 38% increase. In China, the number rose from 133.47 million to 184.75 million, representing a 38% increase. Incident cases In East Asia rose from 11.96 million in 1990 to 13.52 million in 2021, representing a 13% increase. Specifically, in China, the number rose from 11.52 million to 13.05 million, also representing a 13% increase. DALYs cases in East Asia rose from 5.22 million to 7.25 million, representing a 39% increase, while in China, they rose from 5.03 million to 6.99 million, also representing a 39% increase. China, accounting for approximately 85% of the total population in East Asia, mirrors the regional trends in disease burden. The EAPC for incidence rate, prevalence rate, and DALYs rate in East Asia were 0.19 (95% CI: 0.19–0.19), 0.23 (95% CI: 0.23–0.23), and 0.22 (95% CI: 0.22–0.23) respectively, while in China, they were 0.20 (95% CI: 0.19–0.20), 0.24 (95% CI: 0.23–0.24), and 0.23 (95% CI: 0.23–0.23), indicating a significant rise in disease burden in both East Asia and China.

### 3.2 SDI regional level

In 2021, the highest numbers of prevalent, incident, and DALY cases of migraine were observed in regions with middle SDI, reaching 371941648.1 (95% CI: 319831620.3–426895953.9), 28437798.3 (95% CI: 25055734.6–32051775.7), and 13921317.3 (95% CI: 2023382.9–30,544,094) respectively. These cases accounted for approximately 30% of the global total. In 2021, the ASPR, ASIR, and ASDR of migraine were highest in high SDI regions (Tables 1–3). The prevalence, incidence, and DALYs rates were 15365.1, 1222.5, and 573.6 per 100,000 people, respectively. Significant percentage changes were observed in the number of prevalent and incident cases in Low-middle SDI regions, but the prevalence and incidence rates showed a downward trend. The largest percentage changes were seen in low SDI regions (around 130–140%), while the smallest changes occurred in high SDI regions (around 11–23%). Notably, from 1990 to 2021, middle SDI regions experienced rapid growth in prevalence, incidence, and DALY rates, with EAPC of 0.18 (95% CI: 0.16–0.19), 0.13 (95% CI: 0.06–0.2), and 0.17 (95% CI: 0.15–0.19) respectively. Additionally, in 2021, middle SDI regions exhibited relatively higher prevalence, incidence, and DALYs. Therefore, middle SDI regions have the highest number of migraine cases, with the fastest growth rate compared to 1990, resulting in the highest EAPC for these rates.

### 3.3 Factors influencing the EAPC in the burden of migraine disease

**Figure 2:**
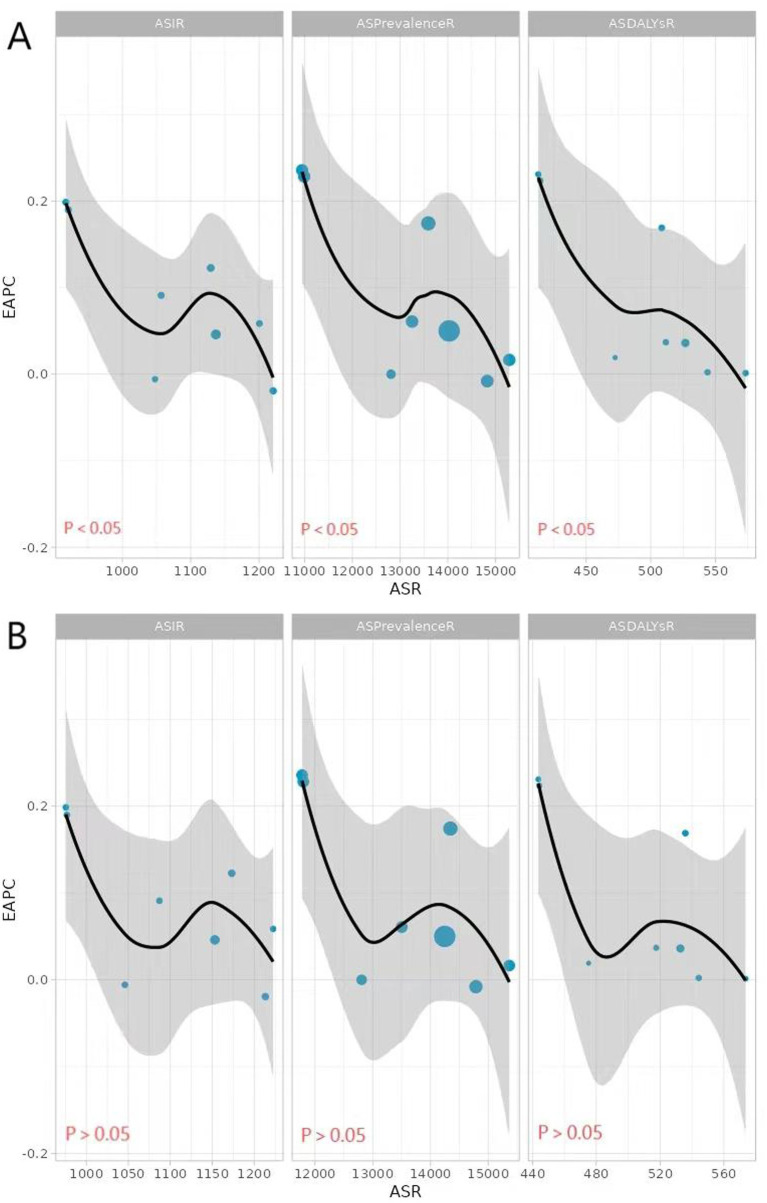
Correlation of ASIR, ASPR, and ASDR for migraine with EAPC in 1990 and 2021 (**A**: 1990, **B**: 2020, from left to right, ASIR, ASPR, and ASDR).

To better interpret the GBD2021 for migraine, this study analyzed factors that could potentially affect the EAPC, including ASIR, ASPR, and ASDR (Figure 2). As shown in Figure 2A, the relationship between EAPC and ASR is not a simple linear correlation. In 1990, there was a significant positive correlation between EAPC and ASIR, ASPR, and ASDR (*r* = 0.5872, 0.6385, 0.6731, *p*-values <0.05). However, in 2021, although there was a moderate correlation between EAPC and ASR (*r* = 0.4128, 0.4557, 0.4968), the *p*-value was greater than 0.05, indicating that this correlation was not statistically significant (Figure 2B).

### 3.4 The correlation between the burden of migraine and SDI

**Figure 3:**
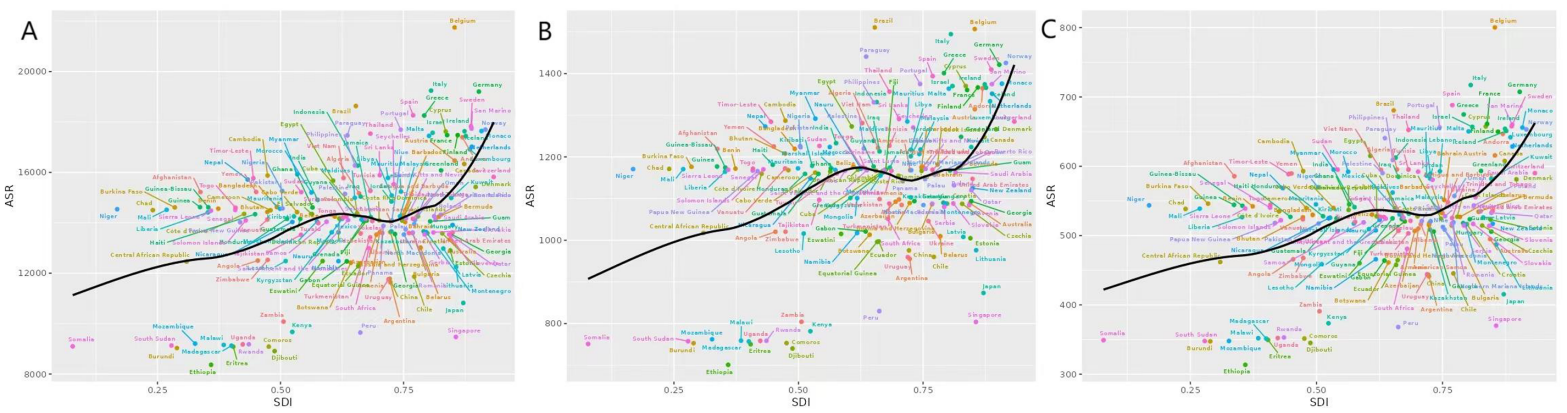
ASPR, ASIR, and ASDR by SDI of migraine in 204 countries and regions (**A**: ASPR, **B**: ASIR, and **C**: ASDR).

In 2021, a positive correlation was observed between the prevalence, incidence, and DALY rates of migraine and the SDI. As economic conditions improved, the overall disease burden showed an upward trend. Specifically, when the SDI value ranged from 0.625 to 0.75, the ASIR burden of migraine exhibited a decline, followed by a significant increase in disease burden alongside rising SDI. Similarly, the ASPR demonstrated a downward trend when the SDI value was between 0.625 and 0.7, with a subsequent continuous escalation in disease burden. The ASDR also showed a downward trend when the SDI value was between 0.6 and 0.7. Notably, the ASIR burden of migraine exceeded expectations in Belgium, Italy, Spain, Brazil, and Paraguay, while it was lower than anticipated in Somalia, Djibouti, Ethiopia, and Singapore (Figure 3).

### 3.5 Frontier analysis of ASPR, ASIR, and ASDR for migraine in 2021

**Figure 4:**
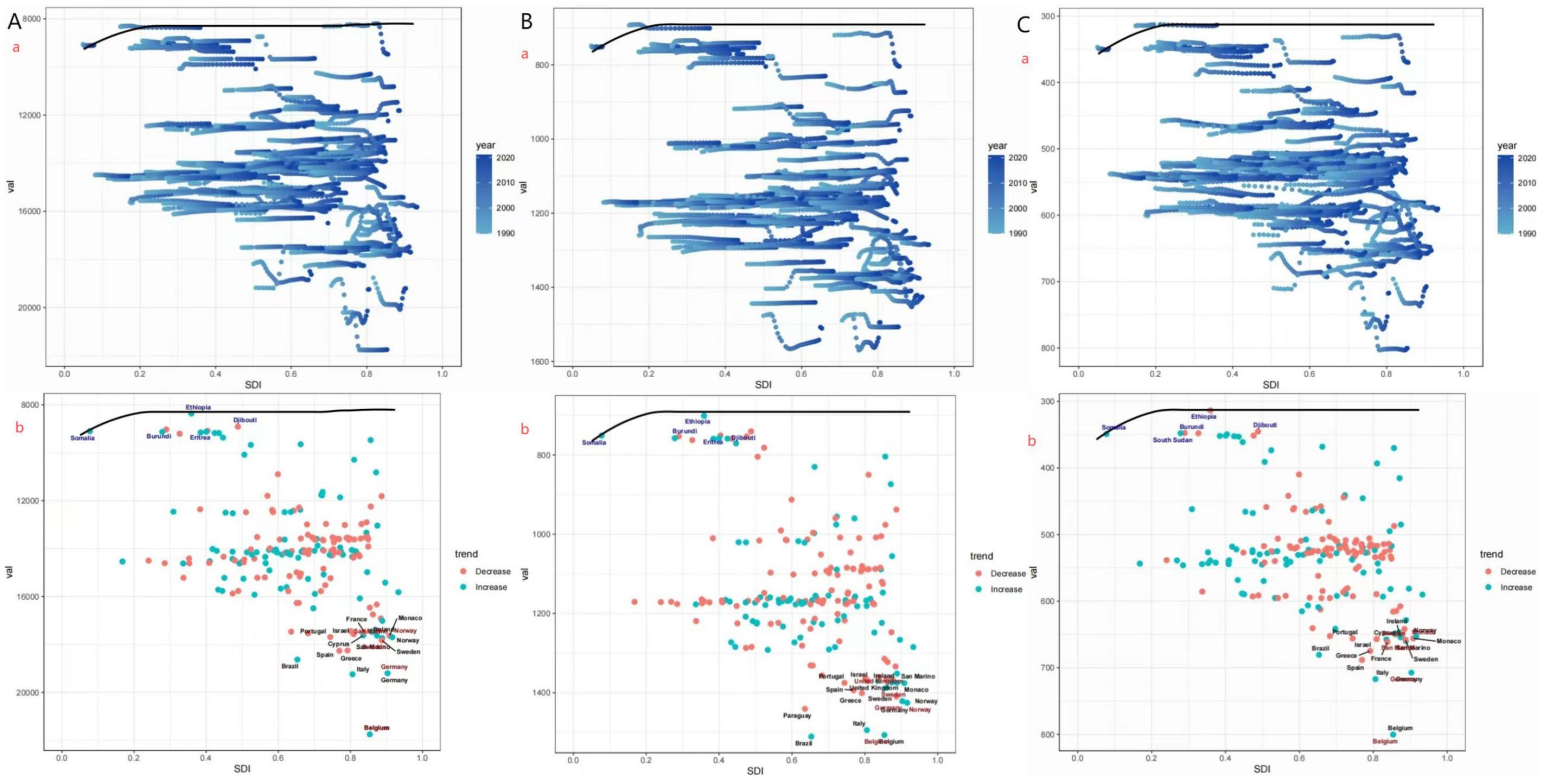
Frontier analysis of ASPR, ASIR, and ASDR for migraine based on SDI in 2021 (**A**: ASPR, **B**: ASIR, and **C**: ASDR).

This study employs frontier analysis to illustrate the health gains across countries or regions with varying levels of development from 1990 to 2021. The findings reveal the unrealized health gains in countries or regions due to various reasons (such as policies, health systems, environmental factors, etc.) that have prevented them from fully utilizing their potential and achieving optimal health improvements. In 2021, the ASPR, ASIR and ASDR burden, as well as effective disparities, progressively increased with the SDI development across countries or regions with different sociodemographic levels (Figure 4). Generally, as sociodemographic development progresses, effective disparities among countries or regions have widened, indicating that countries or regions with a higher SDI have greater potential for burden improvement. There is still room for improvement in achieving health equity, effectively implementing disease prevention and management, and enhancing the quality of health services.

### 3.6 Future predictions of the global disease burden of migraine

**Figure 5:**
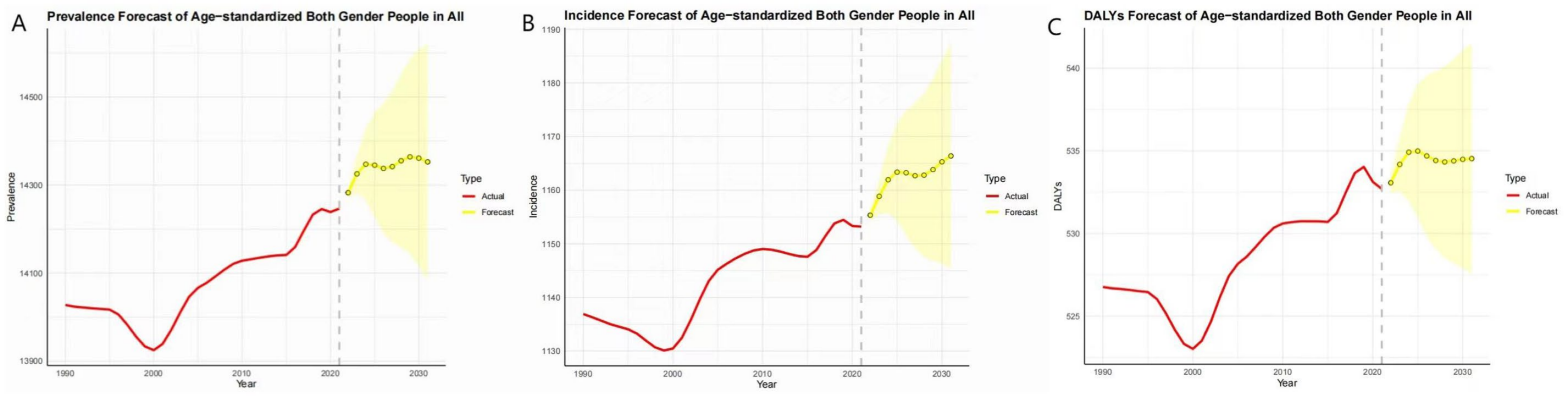
Trend of ASPR, ASIR, and ASDR for migraine from 2021 to 2031 (**A**: ASPR, **B**: ASIR, and **C**: ASDR).

This study presents the predicted trends for the future burden of migraine. The findings reveal an upward trajectory in the ASPR, ASIR, and ASDR of migraine globally. Specifically, the ASPR is expected to rise significantly from 2022 to 2025, followed by a steady but continued increase between 2025 and 2031 (Figure 5A). Similarly, the ASIR demonstrates a notable upward trend from 2022 to 2025, with a period of stabilization between 2025 and 2028, subsequently resuming its upward climb (Figure 5B). Regarding the ASDR, a significant increase is anticipated from 2022 to 2025, followed by a slight decrease and then a leveling off between 2028 and 2031 (Figure 5C).

## 4 Discussion

Our analysis reveals distinct patterns of migraine burden across the socioeconomic spectrum. While high-SDI regions exhibited the highest ASIR, ASPR, and ASDR, it is the middle-SDI regions that emerge as the epicenter of the growing epidemic, bearing the highest absolute number of cases and demonstrating the most rapid growth in ASRs. This divergence highlights the complex interplay between development, healthcare access, and disease risk. This pattern of increasing burden in developing regions aligns with the concept of the ‘fourth stage of epidemiologic transition’, characterized by the rising prominence of non-fatal but disabling chronic conditions as societies develop (9). Our findings that migraine burden escalates specifically in middle-SDI settings are consistent with broader empirical evidence of complex health transitions in low- and middle-income countries (10). This transition is particularly evident for neurological disorders, which are now recognized as a leading and growing cause of disability worldwide, especially in developing regions (11). The rapid urbanization and adoption of lifestyle-related risk factors in these transitioning economies, which are well-established drivers of chronic disease mortality and morbidity (12), likely represent key mechanisms underlying the observed rise in migraine incidence and burden. However, ASIR and ASPR showed a downward trend in low and low-middle SDI regions. Despite the sharp increase in global migraine cases over the past 31 years, ASIR, ASPR, and ASDR did not demonstrate a rapid growth trend. This slow growth is consistent with our findings for the EAPC.

SDI is a composite index based on the total fertility rate of women under 25, lag-distributed income, and average education level among those aged 15 and above (13). Our study suggests that regions with high SDI levels have the highest ASR for migraine, while middle SDI regions bear the highest number of cases, EAPC, and percentage change in case counts. This could be attributed to the rapid urbanization and modernization processes in middle-income regions, accompanied by higher work stress, faster-paced lifestyles, and rapid changes in the social environment. These factors include sedentary lifestyles, stress, lack of physical activity, excessive use of medications and electronic devices, and deteriorating sleep patterns (14). Additionally, with the improvement of healthcare systems in middle-income countries, the diagnosis rate of migraine has increased. Enhanced public health awareness has led to greater recognition and acceptance of migraine, resulting in more people seeking medical help and consequently, an increase in diagnosed migraine cases (15). This contributes to the rise in migraine prevalence, case counts, and ASR. Furthermore, our study observed significant growth in the number of affected and incident cases in low-middle and low SDI regions, but the ASPR, and ASIR declined. This trend may be associated with the increasing proportion of the older adult population in these regions (16). It is worth noting that changes in ASR and EAPC in low, low-middle, and middle SDI regions are expected to impose a growing burden on individuals and society due to population growth and rising global SDI levels.

Furthermore, the COVID-19 pandemic has acted as a major disruptor, likely exacerbating both the global burden of migraine and inequities in its management. Healthcare resources worldwide were diverted toward pandemic response, leading to widespread interruptions in non-urgent medical services. A study conducted in Spain detailed severe disruptions to headache care, including cancellation of outpatient visits, delays in diagnosis, and interruptions in essential treatments (17). Global surveys by the World Health Organization further confirm that such service disruptions were particularly acute in resource-limited settings, with low and middle-income countries experiencing more severe and prolonged impacts on non-urgent medical care (18). Consequently, the pandemic has probably contributed to substantial underdiagnosis, which masks the true incidence of the disease, and has worsened management of existing cases. The lack of timely and standardized treatment likely increased patient disability, a effect ultimately reflected in rising disability-adjusted life years (DALYs). The rapid adoption of telemedicine, while serving as a critical solution, has exhibited substantial disparities in effectiveness across different Socio-demographic Index (SDI) settings, thereby creating a “digital divide” (19). Barriers to access, which include geographical isolation, socioeconomic disadvantage, and inadequate digital infrastructure (20, 21), have posed particularly severe challenges for patients with significant physical or mental disabilities (22). Thus, the pandemic has intensified the current management challenges and underscored the imperative for establishing more resilient and equitable healthcare systems.

The notably rapid increase in migraine burden in middle-SDI regions, as highlighted by our study, likely stems from a dual dynamic: a genuine rise in incidence coupled with major improvements in case ascertainment. The improvement in ascertainment can be primarily attributed to enhanced financial capacity resulting from socioeconomic development, which directly addresses the predominant structural barrier to healthcare access in resource-limited settings, namely financial difficulty (23, 24). Empirical evidence from India provides robust support for this mechanism, demonstrating that a health insurance scheme for the impoverished significantly increased outpatient service utilization (25). Consequently, as economic barriers are removed, patient behavior shifts decisively from endurance to active healthcare use. This behavioral shift, reinforced by growing health awareness, fundamentally alters care-seeking patterns for conditions like migraine (26). Leading to the identification of a substantial pre-existing burden. Concurrently, health system strengthening in these regions, particularly investments in primary health care, has improved the diagnostic capacity for chronic diseases at the grassroots level. Migraine is recognized to be substantially under-diagnosed globally, and the World Health Organization has explicitly identified training primary care physicians as a key strategy to address this problem. Studies confirm that introducing diagnostic guidelines and tools in primary care settings can effectively correct the under-recognition and misdiagnosis of headache disorders (27). Together, these factors systematically uncover a substantial pre-existing burden of migraine that was previously overlooked by the healthcare system.

Simultaneously, the rapid urbanization and lifestyle transitions characteristic of middle-SDI regions represent a contributing factor to a true increase in migraine incidence. World Health Organization reports indicate that urbanization promotes increased risk for various non-communicable diseases. Within this epidemiological shift, increased population exposure to psychosocial stress, sedentary behavior, and dietary changes, all of which are established as modifiable risk factors for migraine (28, 29), is likely instrumental. Consequently, the observed escalation in burden likely reflects both improved detection of pre-existing cases and a genuine rise in disease incidence driven by these risk environments, underscoring the dual challenge faced by health systems in transitioning economies.

This study indicates that the prevalence, incidence, and DALY rates of migraine showed an overall upward trend with economic improvement in 2021. Generally, countries with higher SDI tend to have more advanced healthcare systems and higher patient engagement. Additionally, factors such as the transition from traditional to industrialized diets, increased exposure to environmental pollution, and lifestyle changes (e.g., reduced physical activity) may contribute to the rising risk of chronic diseases (30, 31). In countries with higher SDI, the larger proportion of the older adult population may lead to an increased disease burden of migraine. Therefore, regions with higher SDI should prioritize measures to reduce specific risk factors associated with migraine and improve early screening and treatment.

We should focus on frontier countries with lower SDI, as frontier analysis reveals their excellent performance despite limited resources. The practices and models adopted by these countries can serve as valuable references for other resource-limited countries facing heavy burdens. Conversely, some high SDI countries and regions, such as Belgium, Germany, and San Marino, have shown poor performance, indicating that other factors have outweighed the health benefits brought by development. Future research needs to further explore the driving factors in leading countries and the obstacles faced by lagging countries.

The pattern of a markedly rapid increase in migraine burden observed in middle- and high-middle SDI regions, as illustrated by China, is likely generalizable to a broader range of countries undergoing similar developmental transitions. Our analysis pinpoints a turning point at an SDI of approximately 0.75, beyond which the age-standardized rates of migraine resume a pronounced upward trend. This pattern of co-evolution between non-communicable disease risk and developmental level aligns with the broader framework established by the Global Burden of Disease study (13). This SDI threshold can serve as a predictive tool, indicating that countries approaching or newly entering this high-risk range are likely to encounter an increasing burden of migraine. This pattern is particularly relevant for a cohort of nations experiencing sustained socioeconomic growth, including Indonesia, India, Vietnam, Peru, and Colombia. Many of these countries, classified by the World Bank as middle- and upper-middle-income economies (32), represent the next group likely to confront this challenge. These nations embody the epidemiologic transition, which is the characteristic shift in population disease burden that accompanies societal development (33).

## 5 Limitations

This study still faces some limitations. Firstly, our research is based on secondary data analysis from the GBD study. Insufficient registration of migraine cases in certain underdeveloped areas may lead to underestimation, despite the use of robust statistical methods to mitigate this effect. Misdiagnosis or missed diagnosis due to limited health resources remains a possibility. Secondly, data from various countries inevitably leads to quality inconsistencies, such as measurement biases, reporting delays, or disease misclassification. While the predictive model provides a general outline of future trends, it cannot offer precise forecasts. Finally, the issue of GBD data lag arises because current estimates are calculated based on past trends and covariates (34). To address the limitations of the GBD study, more international collaborations should be encouraged, including annual data searches with partners from various countries and the incorporation of case data from a wider range of sources. Additionally, the detailed data cleaning, correction, and smoothing procedures developed by GBD collaborators are effective measures to overcome these limitations. Ultimately, we need to translate research findings into practical actions, such as formulating public policies and providing references for future studies.

## 6 Conclusion

In summary, migraine represents a significant global public health issue. This study has found that the burden of migraine among the global population has increased significantly from 1990 to 2021, particularly in regions with middle SDI. Our research underscores the importance of tailored interventions aimed at addressing migraine, thereby contributing to the achievement of the Sustainable Development Goals set by the World Health Organization. The formulation and implementation of scientifically effective public health policies, along with the enhancement of health education and medical services, are crucial in reducing the burden of migraine.

## Data Availability

The original contributions presented in the study are included in the article/supplementary material, further inquiries can be directed to the corresponding authors.
